# Outcomes of a Multicenter Safety and Efficacy Study of the SuitX Phoenix Powered Exoskeleton for Ambulation by Patients With Spinal Cord Injury

**DOI:** 10.3389/fneur.2021.689751

**Published:** 2021-07-19

**Authors:** Paul Aarne Koljonen, Anna Sternin Virk, Yoon Jeong, Michael McKinley, Juan Latorre, Amaya Caballero, Yong Hu, Yat Wa Wong, Kenneth Cheung, Homayoon Kazerooni

**Affiliations:** ^1^Department of Orthopaedics and Traumatology, Maclehose Medical Rehabilitation Centre, Hong Kong West Cluster, Hospital Authority, Hong Kong, China; ^2^Department of Orthopaedics and Traumatology, The University of Hong Kong Li Ka Shing Faculty of Medicine, Hong Kong, China; ^3^US Bionics at Emeryville, Emeryville, CA, United States; ^4^St. David's Medical Center, Austin, TX, United States; ^5^University of Virginia School of Medicine, Charlottesville, VA, United States; ^6^Department of Mechanical Engineering, University of California, Berkeley, Berkeley, CA, United States

**Keywords:** spinal cord injury, paraplegia, exoskeleton, rehabilitation, robotics

## Abstract

**Objective:** To examine the safety and efficacy of ambulation utilizing a semi-passive and lightweight powered exoskeleton by spinal cord injury (SCI) patients.

**Methods:** This is a multi-center, open-label, prospective cohort study across three facilities. A cohort of 40 individuals with SCI from T4-L5 was recruited into a 20-session training and assessment protocol, utilizing the SuitX Phoenix. All patients were tested using a 10-m-walk test (10 MWT), 6-min-walk test (6 MWT), and Timed up & Go test (TUG). Patient satisfaction, pain, exertion, changes in affect, as well as overall comfort and confidence were reported using a satisfaction survey, Rated Perceived Exertion (RPE) scale, and Positive and Negative Affect Schedule (PANAS). Safety outcomes, adverse events, and device malfunctions were reported.

**Results:** Forty participants completed the study. There were no serious adverse events. All participants reported moderate to high levels of comfort and confidence using the device. All patients were able to achieve FIM of >4 on transitional movements and walking. The neurological level of injury had a statistically significant association with walking speed, WISCI-II, and FIM. Participants with an incomplete spinal cord injury had a higher FIM, faster speed, and higher WISCI-II in all outcome measures.

**Conclusion:** This is the first study to examine the safety and efficacy of SuitX Phoenix for ambulation by SCI patients. We have shown that Phoenix is efficacious in allowing adults with SCI T4 to L5 perform walking and transitional movements. This study also reports the safety-profile of the device, user satisfaction, and psychological trends during training.

## Introduction

The worldwide annual incidence of SCI is estimated to be ~23 per million capita, and in the United States, ~276,000 individuals suffer from spinal cord injuries (SCI). Internationally, another 250,000–500,000 persons are added to the population of SCI patients annually (1).

In the US, paraplegia comprises 40% of all SCI cases. A 2011 longitudinal cohort study reported that 59% of patients with traumatic SCI remained unable to walk 1 year following their injury (2), while one study indicated that the ability to walk again remains an important goal (3).

In addition to helping SCI individuals regain the ability to walk, the rapidly improving capabilities of powered exoskeletons now also provide us with prospects of neurorehabilitation. This concept of neural reconditioning *via* locomotion has evolved from animal and human studies focused on the neural plasticity of the spinal cord (4–6). When guided with repetition and habituation, locomotor training and neuromuscular activation may promote physical and biochemical modification of synaptic connections, thus leading to functional improvements even with incomplete regeneration (7–9).

While powered exoskeletons are generally operated by experienced physical therapists or patients with trained or certified aides, studies have shown that they are generally safe and well-tolerated by patients in both hospital and community settings. Miller et al. reported in a meta-analysis of 14 studies (eight ReWalk, three Ekso, two Indego, and one unspecified exoskeleton) that exoskeletons can allow safe ambulation while maintaining an appropriate physiological intensity conducive for health benefits (10). However, several aspects including cost and applicability to activities of daily living (ADL), often limit the use by patients for training or recreation. Furthermore, most currently available exoskeleton units tend to be heavy, bulky, and costly, which makes the use of devices more inconvenient.

This study is designed to examine the safety and efficacy of a novel lightweight exoskeleton (SuitX Phoenix). To the best of our knowledge, this is also the first study that examines the efficacy of an exoskeleton with semi-passive knee joints.

The primary objective of this study was to evaluate the ability of Phoenix to enable individuals with paraplegia to engage in supervised ambulation and transitional tasks. Based on the primary innervation of abdominal and truncal muscles, we used T7 as a division between two groups of patients with different functional goals. We hypothesize that patients will be able to complete the primary objectives according to the criteria listed in Table 1.

**Table 1:** Study objectives and endpoints.

**Primary objective and endpoint**	**Secondary and tertiary objectives and endpoints**
■ Participants with SCI neurological level T7 to L5 can safely complete transitional movements (stand up, turn, sit down) and walk using Phoenix with minimal contact assistance [Functional Independence Measure (FIM) score 4]. The study will use the 10 Meter Walk Test (10 MWT), Timed Up and Go (TUG), and 6 Minute Walk Test (6 MWT) as methods of assessment for these objectives. ■ Participants with SCI neurological level T4 to T6 can safely complete transitional movements (stand up, turn, and sit down) and walk using Phoenix with moderate physical assistance [Functional Independence Measure (FIM) score 3]. The study will use the 10 Meter Walk Test (10 MWT) and Timed Up and Go (TUG) as methods of assessment for these objectives.	■ Individuals are able to use the Phoenix to walk safely under a variety of conditions using the Surface Walk Test (SWT) on carpet and concrete, with FIM score of not <4. ■ Individuals are able to use the Phoenix to walk with an acceptable level of exertion, as measured by Rated Perceived Exertion (RPE) of 4 or less on the Modified Borg Scale. ■ Individuals are able to walk with the Phoenix without added pain and fatigue, as measured on a Numeric Rating Scale. Expected levels of pain and fatigue before beginning a session are 0, unless there is already baseline pain and fatigue present. Pain levels are not expected to increase after the session, and fatigue levels are expected to stay at 5/10 or less. ■ Any positive or negative alteration in affect during training was captured by the PANAS scale at the beginning and end of the study These are purely observational values which do not have expected scores.

A secondary study objective was to assess the safety and efficacy of individuals using Phoenix to walk under a variety of surface conditions.

A tertiary/exploratory objective was the study of exertion, pain, fatigue, mood, and satisfaction after exoskeleton use. The assessment tools used for this section are numerical pain scales, self-rated perceived exertion (RPE) scales, user questionnaires, as well as the positive and negative affect schedule (PANAS) (11). The criteria for successful study completion is the participant completing all 20 sessions according to protocol.

## Methods

### Study Design

This is a multi-center, open-label, prospective cohort study. Institutional Review Board (IRB) approval was attained at all study sites. Adult spinal cord injury (SCI) participants with neurological levels from T4- L5, who met the inclusion/exclusion criteria (Table 2) were recruited. The clinical study was performed at three sites: US Bionics at Emeryville, CA; St. David's Medical Center at Austin, Texas; and at The Maclehose Medical Rehabilitation Centre in Hong Kong. The trial was registered with clinicaltrials.gov (#NCT NCT03175055) and was designed with the intent to demonstrate safety and feasibility to the US Food and Drug Administration (FDA).

**Table 2:** Inclusion and exclusion criteria.

**Eligibility criteria**	**Exclusion criteria**
■ T4-L5 neurological level of SCI ■ 18 Years of age or older ■ Weigh no more than 200 lbs. ■ Skin must be healthy where it touches the Phoenix ■ Able to stand using a device such as a standing frame ■ Normal myotomal innervation and functional control of upper limbs. ■ Determined to have enough bone health to walk full weight bearing without risk for fracture. Meeting of this condition is at the discretion of the participant's physician ■ Normal functional range of motion of upper and lower limbs. ■ Hip width no greater than 18" (46 cm) measured when sitting ■ Femur length between 12.3 inches (31.3 cm) and 19.8 inches (50.2 cm) measured between centers of hip and knee joints ■ Tibia length between 13.4 inches (33.9 cm) and 22 (55.9 cm) inches measured between the knee joint and bottom of the foot ■ In general, good health and able to tolerate moderate levels of activity ■ Blood pressure and heart rate within established guidelines for locomotive training ° At rest: Systolic 150 mmHg or less, Diastolic 90 mmHg or less, and Heart rate 100 beats per minute or less ■ Exercise: Systolic 180 mmHg or less, Diastolic 105 mmHg or less, and Heart Rate 145 beats per minute or less	■ Pregnant or lactating females ■ Spinal cord injury level higher than T4 ■ Significant spasticity (Modified Ashworth Scale score of 3 or above) ■ Trunk or lower extremity pressure ulcer ■ Open Wounds ■ Unstable spine, un-healed limbs, or fractures ■ Severe sensitivity to touch ■ Presence of bone in soft tissue where bone normally does not exist (heterotopic ossification), limiting range of motion in the hip or knee joints ■ Joint instability, dislocation, moderate to severe hip dysplasia ■ Significant scoliosis (>40°) ■ Hardware, implant, or any external device impeding with safe fitting or use of Phoenix ■ Femoral or tibial rotation deformity (>15°), or other joint deformity which may impede with safe use of the device ■ Significant flexion contractures limited to 35o at the hip and 20o at the knee ■ Uncontrolled seizures, musculoskeletal injury, fracture or lower-limb surgery in past year ■ Known history of pulmonary disease limiting exercise tolerance or history of cardiac disease ■ Known psychological or psychiatric diagnosis which may preclude safe usage of an exoskeleton device. ■ Medical comorbidities such as cardiac or respiratory conditions which preclude safe usage of an exoskeleton device. ■ Dizziness or headache with standing ■ History of autonomic dysreflexia ■ Orthostatic Hypotension: Decrease in Systolic BP > 20 mmHg, Diastolic BP > 10 mmHg upon standing from a seated position

### Description of Device

**Figure 1:**
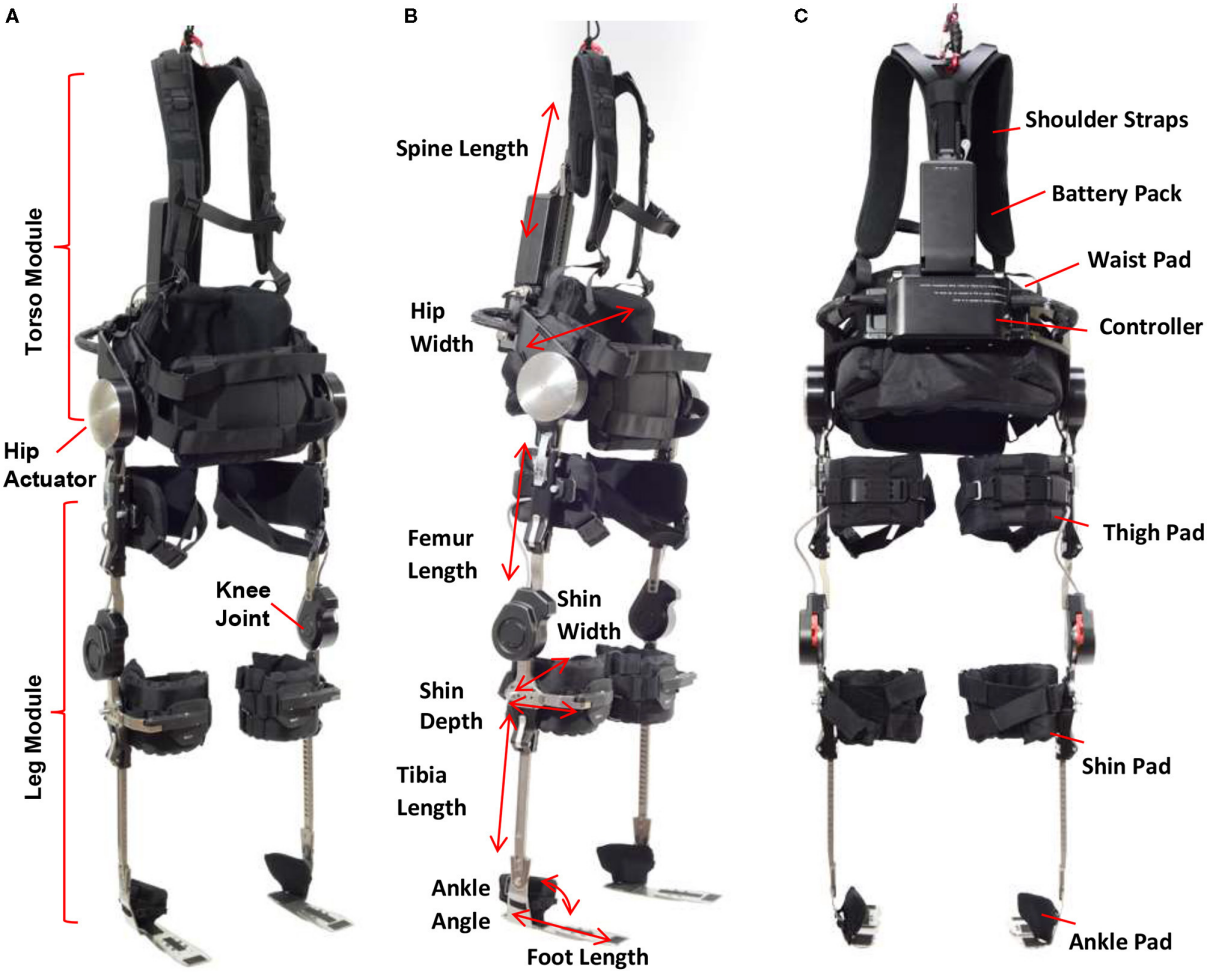
**(A)** Phoenix modules, **(B)** adjustments, **(C)** padding and adjustments.

**Figure 2:**
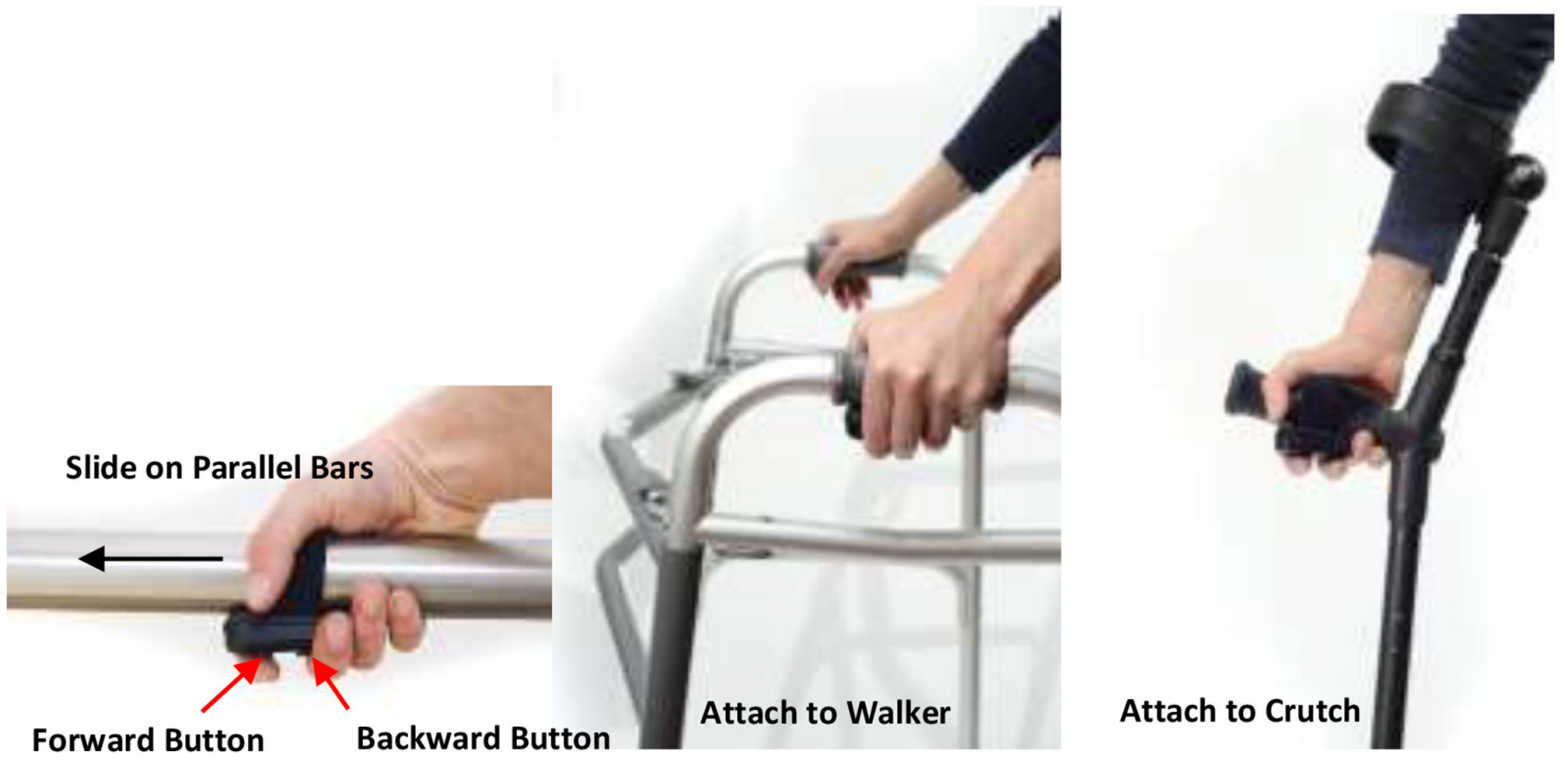
Phoenix user interface.

Phoenix consists of a torso module and two leg modules (Figure 1A). The torso module has two powered actuators located on each side of the hip which are powered by rechargeable batteries located in the torso module. A controller for the device located below the battery pack controls the movement of the hip joints and trigger of knee joints. The leg module contains a knee joint, adjustable tibial link, thigh link, and foot plates that fit inside the wearer's shoes. The knee joint has an electronic gauge to trigger the locking/unlocking actions. The timing of these actions is programmed to provide toe clearance during swing phase, and secure support during stance phase. Parameters related to swing and stance, such as hip flexion angle and speed, are programmed *via* a tablet-based application. The tablet communicates with the device in real-time such that a practitioner can program personalized gait parameters for each individual during training or gait therapy. While the tablet serves as a practitioner's interface to Phoenix to tune parameter, the exoskeleton wearer controls the device to take steps, sit down, and stand up *via* a handheld user interface. The handheld user interface (Figure 2) can be mounted on a handle of a crutch, walker, or a parallel bar rail depending on the stage of training. The user interface has two buttons; a “forward” button to command progressing actions such as (1) standing up from sitting, (2) taking a step from standing, and a “backward” button to command returning actions such as (1) standing with feet together from walking, (2) sitting down from standing.

The height and width of the torso component as well as the length of tibia and femur components can be adjusted to accommodate the wearer's size and proportions. Additionally, shoulder straps, tibial, thigh and ankle pads, and a torso pad can be adjusted to provide comfort and prevent skin abrasion while using the device (Figures 1B,C). Phoenix weighs 15 kg including batteries.

**Figure 3:**
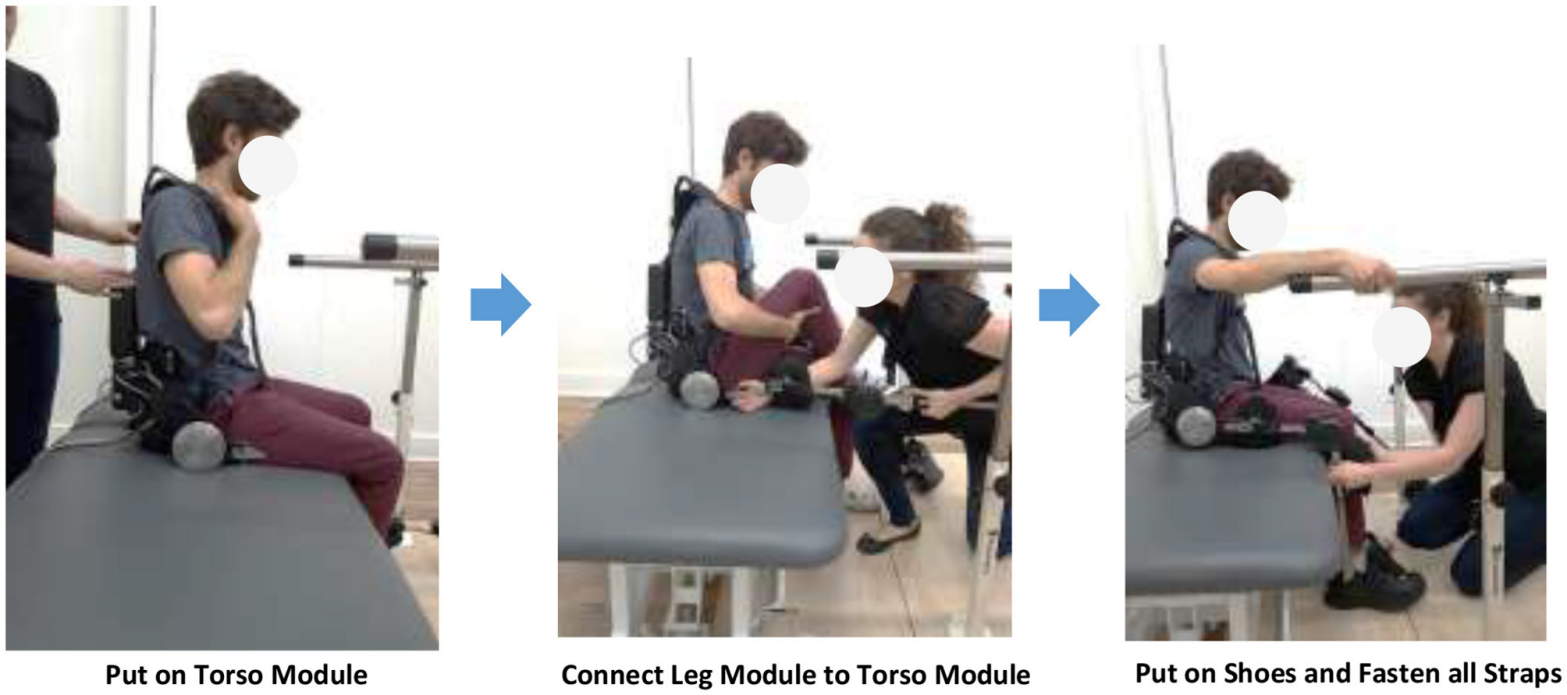
Putting on the Phoenix exoskeleton.

When the size of the device is adjusted to the user, the user can put on the device by first wearing the shoulder straps of the torso module, connect the torso module and leg modules, put on shoes, and fasten all straps. The user may put on or take off the device with or without the help of a practitioner (Figure 3).

### Training Protocol

During this study, participants were trained to use the Phoenix Exoskeleton throughout 20 supervised 1 h-sessions. All sessions were conducted by the principal investigator or a designated clinical investigator for each site.

All investigators were trained to conduct the Phoenix Clinical Training Program, which included an in-depth overview donning/doffing, device operations, and gait parameter adjustments. Sessions proceeded through an IRB-approved protocol which is summarized in Table 3. The sessions included training for donning (putting on) and doffing (taking off) of the exoskeleton, proficiency in sit-to-stand and vice versa, weight-shifting, parallel bar walking, frame/crutch walking, turning, and various surface walking.

**Table 3:** Outline of training and assessment protocol.

**Process**	**Activity**	**Forms**
Pilot application	Forms completed by candidate and MD	Phoenix trial application Participant screening survey
Phone pre-screening	Video call to check ROM or Personal visit to test facility	Participant tele-interview or on-site interview
Screening and baseline testing	PT evaluation and measurements 10 MWT, TUG, 6 MWT, SWT, WISCI II	Consent to participate; daily protocol session 1, medical history, PANAS
Training	Learn to use the device: pre-gait and gait activities	Daily protocol sessions 2–10
Mid-point outcome testing	10 MWT, TUG, 6 MWT, TUG	Daily protocol session 11
Training	More practice on pre-gait and gait activities	Daily protocol sessions 12–18
Final outcome testing	10 MWT, TUG, 6 MWT, TUG, WISCI II	Daily protocol sessions 19–20, test participant survey, PANAS

We performed all primary testing on flat surfaces such as indoor gymnasium areas or hospital hallways. The surface walk test was performed on carpet and concrete surfaces. All flat surfaces were <2° in slope with no obstacles. The intended use environments of the Phoenix in this study were clinical and non-clinical indoor settings of rehabilitation facilities.

### Assessment Methods

Study staff performed meticulous assessments of skin integrity at the start and end of each session in order to ensure there were no pressure injuries at locations coming into contact with the exoskeleton. They also recorded vital signs, pulse oximetry, pain, fatigue, and exertion levels at the beginning and end of each session. A numeric rating scale was used for pain, and a rated perceived exertion index (BORG scale) was used for recording fatigue and exertion levels. Formal patient assessments were performed during sessions 1, 11, 19, and 20.

#### Evaluation Indices

Several standard indices for evaluating levels of independence for ambulation by individuals with paraplegia were adopted in our study. These include:

1. Functional Independence Measure (FIM) – the 7-point ordinal scale for the motor component was used for the study, with one representing total assistance and seven representing complete independence. A score of four indicates that the subject could complete 75% or more of the task with minimal assistance.
2. Walking Index for Spinal Cord Injury (WISCI-II) – an ordinal scale from 1 to 20 capturing the extent and nature of assistance (combinations of orthoses, supporting equipment such as walkers and human helpers), with a higher score indicating increased levels of independence.
3. 10 m walk test (10 MWT)—A performance measure used to assess walking speed in meters per second over a 10-m distance.
4. 6 min walk test (6 MWT)—A performance measure used to assess walking distance in meters over a 6-min testing time.
5. Timed up-and-go (TUG)—A performance test used to assess a person's mobility and requires both static and dynamic balance. It measures the time that a person takes to rise from a chair, walk 3 m, turn around 180°, walk back to the chair, and sit down while turning 180°.

The psychological changes of subjects undergoing training were also serially documented using the Positive and Negative Affect Schedule (PANAS) at sessions 1, 11, 19, and 20. Furthermore, after the last study session, participants completed test participation surveys which included questions on subjective assessment of the fitting and adjustment process, ease/difficulty of training, pain and fatigue, respiratory problems, and bowel/bladder movement.

Vitals, adverse events, unanticipated problems, and device malfunctions were recorded during all sessions and appropriately resolved. All device inadequacies relating to quality, durability, reliability, safety, or performance were documented throughout the study and reported. Protocol deviations were meticulously documented with justifications.

### Statistical Analyses

An independent biostatistician unaffiliated with SuitX performed all statistical analyses. Descriptive statistics included baseline sociodemographic and injury characteristics. Associations between continuous clinical variables were studied using one-way ANOVA. The mean FIM for the 10-MWT, 6-min-walk-test, TUG, and surface testing was stratified by study site. The mean WISCI-II scores were also stratified by study site. Trends in primary endpoints throughout the study period were assessed using one-way ANOVA to compare scores between baseline, midline, and final study sessions. Correlations of walking speed with age, sex, and neurological level were performed using Pearson correlation. Frequency tables of adverse events, unanticipated problems, malfunctions, compliance, and enrolment figures were generated. PANAS scores were assessed over time as well as assessed at the end of the study by comparing levels between study sites using one-way ANOVA. Pain, fatigue, and exertion levels were compared pre-and post-session as well as over time by assessing session 1, session 19, and session 20 scores. Trends in pain, fatigue, and exertion levels were compared in two ways: (1) by comparing all pre-session scores with all post-session scores using a student *t*-Test, and (2) by comparing scores from session 1 to session 20 stratified by whether assessments were pre- or post-session by using ANOVA.

## Results

### Patient Demographics

The study enrolled a total of 61 participants, and 40 completed the study. All participants were chronic SCI individuals (over 1-year post-injury). Twenty-one participants did not complete the study: six screening failures—i.e., participant passed screening, but study staff identified inclusion/exclusion criteria were not met after Session 1, two voluntary withdrawals, five lost to follow-up, and eight staff withdrawals—i.e., participant was unable to comply adequately with the study protocol. Table 4 shows a summary of baseline characteristics. Besides ethnicity, there were no significant differences in the baseline characteristics of participants across three sites.

**Table 4:** Patient characteristics and demographics.

**Characteristic**	**SITE 1 – Suitx**	**SITE 2 - Hong Kong**	**SITE 3 - St. David's**
	***n* (% cohort)**	***n* (% cohort)**	***n* (% cohort)**
**Sex**			
Male	15 (75.0%)	10 (66.7%)	3 (60.0%)
Female	5 (25.0%)	5 (33.3%)	2 (40.0%)
**Ethnicity**			
White, Non-Hispanic	13 (65.0%)	1 (6.7%)	5 (100.0%)
Hispanic	5 (25.0%)	0 (0%)	0 (0%)
Asian	0 (0%)	13 (86.7%)	0 (0%)
Other	2 (10.0%)	1 (6.7%)	0 (0%)
**Injury Level**			
T4-T9	8 (40.0%)	7 (46.7%)	4 (80.0%)
T10-T12	10 (50.0%)	5 (33.3%)	0 (0.0%)
L1-L3	2 (10.0%)	3 (20.0%)	1 (20.0%)
**ASIA Score**			
A	8 (40.0%)	12 (80.0%)	4 (80.0%)
B	5 (25.0%)	0 (0%)	0 (0%)
C-D	7 (35.0%)	3 (20.0%)	1 (20.0%)

### Primary Study Outcome

Table 5 displays the results for primary outcomes. At all study sites, the mean FIM for the 10-m-walk-test, 6-min-walk-test, timed-up-and-go, and on all surfaces walk tests was above four. In a *post-hoc* analysis of walking performance as a function of the neurological level of injury and severity of SCI, we found that both had a statistically significant association with several endpoints. Although we identified that level of SCI had a statistically significant association with 10 MWT FIM score, walking speed, 6-min-walk-test FIM score, WISCI-II score, carpet and concrete FIM score, only the WISCI-II and walking speed demonstrated a clear trend (lower injury level performed better) (Table 6). Similarly, whether the participants' spinal cord injury was complete vs. incomplete showed a statistically significant association with FIM scores on all surfaces, walking speeds, and WISCI-II scores. With incomplete SCI patients performing better on all the above (Table 7).

**Table 5:** Functional outcome summary.

**Outcome**	**SITE 1 - Suitx** **(*n* = 20)**	**SITE 2 - Hong Kong (*n* = 15)**	**SITE 3- St. David's (*n* = 5)**	**Overall (*n* = 40)**
	**Mean (+/-SD)**	**Mean (+/-SD)**	**Mean (+/-SD)**	**Mean (+/-SD)**
10 MWT FIM	4.8 (±0.41)	4.2 (±0.41)	4.6 (±0.55)	4.6 (±0.50)
10 MWT Speed (m/s)	0.11 (IQR: 0.08–0.14)	0.11 (IQR: 0.07–0.13)	0.10 (IQR: 0.08–0.13)	0.11 (IQR: 0.08–0.14)
6 MWT FIM	4.63 (±0.50)	4.14 (±0.36)	4.00 (±0)	4.37 (±0.49)
TUG FIM 20	4.15 (±0.37)	4.07 (±0.46)	4.20 (±0.44)	4.13 (±0.40)
WISCI-II 19	9.45 (±1.93)	8.07 (±2.31)	6.80 (±1.30)	8.60 (±2.19)
Carpet FIM	4.80 (±0.41)	4.20 (±0.41)	4.60 (±0.55)	4.55 (±0.50)
Concrete FIM	4.60 (±0.50)	4.90 (±0.31)	4.20 (±0.41)	4.60 (±0.55)
BORG session 19 (10 MWT, SWT)	3.45 (±1.47)	2.67 (±1.23)	3.80 (±2.39)	3.20 (±1.54)
BORG session 20 (6 MWT, TUG)	3.33 (±1.37)	2.67 (±1.23)	4.40 (±1.52)	3.33 (±1.37)

*At all study sites, the mean FIM for the 10-m-walk-test, 6-min-walk-test, timed-up-and-go, and on all surfaces was >4*.

**Table 6:** Stratification of outcome metrics according to the neurological level of spinal cord injury three groups (T4-9, T10-12, L1-3).

**Outcome**	**T4-T9 (*n* = 19)**	**T10-T12 (*n* = 15)**	**L1-L3 (*n* = 6)**	***p***
10 MWT FIM	4.53 (±0.51)	4.60 (±0.51)	4.50 (±0.55)	0.04
10 MWT Speed (m/s)	0.11 (±0.06)	0.12 (±0.04)	0.15 (±0.07)	0.01
10 MWT Time (s)	73.00 (±43.26)	55.07 (±20.36)	50.50 (±26.55)	0.03
6 MWT FIM	4.18 (±0.39)	4.53 (±0.52)	4.50 (±0.55)	0.03
TUG FIM session 20	4.11 (±0.46)	4.07 (±0.26)	4.33 (±0.52)	0.43
WISCI-II session 19	8.05 (±1.93)	9.0 (±2.17)	9.33 (±2.94)	0.01
Carpet FIM	4.53 (±0.51)	4.53 (±0.52)	4.67 (±0.52)	0.04
Concrete FIM	4.52 (±0.51)	4.67 (±0.49)	4.67 (±0.52)	0.03
BORG session 19	3.53 (±1.90)	3.13 (±0.92)	2.33 (±1.37)	0.97
BORG session 20	3.63 (±1.42)	3.13 (±0.92)	2.83 (±2.04)	0.85

*The level of SCI had a statistically significant association (p < 0.05) with 10 MWT FIM score, 10 MWT speed, 10 MWT time to completion, 6 min-walk-test FIM score, WISC-II score, carpet, and concrete FIM score*.

**Table 7:** Stratification of outcome metrics according to the severity of spinal cord injury (complete vs. incomplete neurological injury).

**Outcome**	**Complete (*n* = 24)**	**Incomplete (*n* = 16)**	***P***
10 MWT FIM	4.42 (±0.10)	4.75 (±0.11)	0.04
10 MWT Speed (m/s)	0.10 (±0.008)	0.15 (±0.02)	0.01
10 MWT Time (s)	72.42 (±8.00)	48.63 (±5.03)	0.03
6 MWT FIM	4.23 (±0.43)	4.56 (±0.51)	0.03
TUG FIM session 20	4.17 (±0.48)	4.06 (±0.25)	0.43
WISCI-II session 19	7.88 (±1.80)	9.69 (±2.33)	0.01
Carpet FIM	4.42 (±0.50)	4.75 (±0.45)	0.04
Concrete FIM	4.46 (±0.51)	4.81 (±0.40)	0.03
BORG session 19	3.20 (±1.69)	3.20 (±1.33)	0.97
BORG session 20	3.29 (±1.30)	3.38 (±1.50)	0.85

*Whether the spinal cord injury was complete vs. incomplete showed a statistically significant association (p < 0.05) with 10 MWT FIM scores, 10 MWT speed, 10 MWT time to completion, 6 min-walk-test FIM scores, WISC-II scores, carpet, and concrete FIM scores*.

There was no significant correlation between speed and age (*R* = 0.015), height (*R* = −0.02) or weight (*R* = 0.06). Walking speed was similar in male (mean 0.114 m/s; SD 0.053) and female participants (mean 0.129 m/s; SD 0.061).

### Results on Pain, Fatigue, Exertion, and Mood

Participants did not show significant change in positive or negative affect from baseline, midpoint, to final assessments. However, positive affect scores displayed a trend upwards, while negative affect scores displayed a trend downwards. These trends were not statistically significant. Table 8 displays the mean PANAS scores which were assessed at baseline, midline (session 11), and during the final sessions (session 19 and 20).

**Table 8:** Changes in subjects' emotional status throughout the entire training protocol using positive and negative affect scale (PANAS).

**Outcome**	**Baseline (*n* = 39)**	**Mid (*n* = 40)**	**Final (*n* = 38)**	***p***
PANAS positive affect score	40.90 (±7.52)	41.26 (±6.86)	42.30 (±6.58)	0.65
PANAS negative affect score	14.05 (±4.20)	13.54 (±4.32)	12.63 (±4.00)	0.31

*Participants did not show a statistically significant improvement in positive or negative affect from baseline, midline, to final assessments. However, PANAS for positive affect scores displayed a trend upwards, while PANAS scores for negative affect displayed a general trend downwards*.

**Figure 4:**
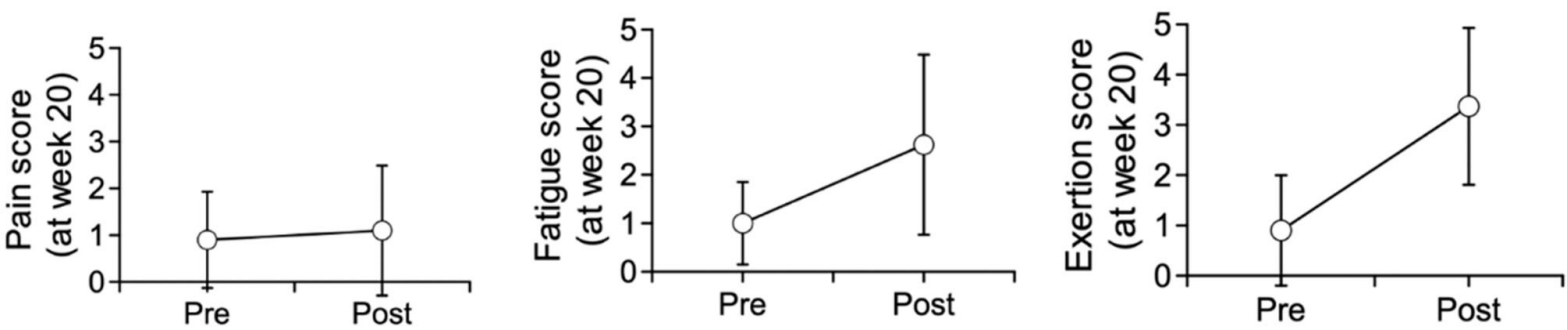
Final session evaluation on pain, fatigue, and exertion trends before and after training.

As displayed in Table 9 and Figure 4, for all sessions, fatigue and exertion levels increased in the post-session period (*P* < 0.001). Pain levels, on the other hand, displayed no significant difference between pre- and post-session.

**Table 9:** Trends in pain, fatigue, and exertion level throughout the entire training protocol by comparing all pre-session scores with all post-session scores (*p*-values displayed using student *T*-test).

**Session**	**Pain (*****p*** **= 0.61)**		**Fatigue (*****p*** **< 0.001)**		**Exertion (*****p*** **< 0.001)**	
	**Pre (Mean +/− SD)**	**Post (Mean +/− SD)**	**Pre (Mean +/− SD)**	**Post (Mean +/− SD)**	**Pre (Mean +/− SD)**	**Post (Mean +/− SD)**
Session 1	1.45 (±1.68)	1.14 (±1.64)	1.05 (±1.15)	1.51 (±1.24)	0.70 (±0.88)	2.35 (±1.69)
Session 11	1.08 (±1.33)	1.28 (±1.52)	1.00 (±1.41)	3.03 (±2.06)	0.80 (±1.32)	3.71 (±1.74)
Session 19	0.88 (±0.97)	1.10 (±1.35)	0.78 (±0.80)	2.31 (±1.59)	0.80 (±0.91)	3.21 (±1.59)
Session 20	0.90 (±1.03)	1.10 (±1.39)	1.00 (±0.85)	2.62 (±1.86)	0.90 (±1.10)	3.37 (±1.56)

For post-session assessments, there was no significant trend in pain levels (*p* = 0.95) from session 1 to 20. However, both post-session fatigue (*p* = 0.002) and exertion (*p* = 0.004) displayed a significant difference between session 1, session 11, session 19, and session 20. Although we did not identify a clear trend, both fatigue and exertion appeared to first display a large increase, followed by a minor decrease, and another minor increase.

### Safety Evaluation and Adverse Events

There were 2 adverse events reported for the entire study, both relating to bruising in the sacral region, and were addressed immediately by pausing the session and adjusting the amount of padding. Less than 10% (9.5%: 91/956) of sessions had a reported mild alteration in skin integrity, such as temporary redness/blanching on shins, heels, or skin overlying foot/ankle bones. These resolved spontaneously without additional treatment required.

Less than 4% (3.2%: 31/956) of all post-session observations included a safety concern that was not severe enough to qualify as an adverse or serious adverse event. The most common subjective complaint was pain/discomfort 74.2% (23/31), followed by fatigue/overexertion 22.6% (7/31), and one episode (3.2%: 1/31) of urinary incontinence. We did not find a statistically significant difference in number of safety concerns between three sites.

### Protocol Deviations

Protocol deviations were meticulously recorded. There was no statistically significant difference between types of deviation and the study site where it occurred. Missing or erroneous data entry was most common, followed by administrative issues with data collection (missing signature, date entry, etc). All deviations were immediately rectified after identification, with appropriate justifications recorded (Table 10).

**Table 10:** Protocol deviation summary.

**Deviation**	**SITE 1 - Suitx (*n* = 25)**	**SITE 2 - Hong Kong (*n* = 34)**	**SITE 3 - St. David's (*n* = 17)**
Missing or erroneous data	14 (56.0%)	18 (52.9%)	12 (70.6%)
Consent form deviations	0 (0%)	4 (11.8%)	1 (5.9%)
Missed sessions	0 (0%)	2 (5.9%)	0 (0%)
Session objective not achieved	1(4.0%)	3 (8.8%)	2 (11.8%)
Administrative issues with data collection	4 (16.0%)	4 (11.8%)	1 (5.9%)
Session protocol deviations	5 (20.0%)	2 (5.9%)	1 (5.9%)
Missed surface testing	1 (4.0%)	1 (2.9%)	0 (0%)

*Protocol deviations are documented and stratified by study site. No statistical significance between the type of protocol deviation and study site was observed (p = 0.47). Missing or erroneous data was most common, followed distantly by administrative issues with data collection and session protocol deviations*.

## Discussion

### Reported Efficacy of Exoskeletons

Personal-use exoskeletons now provide individuals with paraplegia with the tools to increase their life-long over-ground walking capacity. This benefit not only reduces complications resulting from prolonged immobilization, but also allows the user to reap the benefits of improved upper body muscular fitness, improved circulatory responses, improved bowel movement regularity, and reduced pain and spasticity (12–15). Increasing exposure to gravitational and muscular loading forces may also minimize decline in bone mineral density (12). As a means of exercise rehabilitation for individuals with SCI, Aach et al. demonstrated significant improvements in gait speed and endurance with the use of a powered exoskeleton (16). Furthermore, the capability of the central nervous system to retrain itself *via* neuroplasticity has presented us with potential to help patients regain meaningful functional reorganization of neural networks *via* guidance with repetition and habituation (17–20).

Unfortunately, one of the barriers for chronic SCI individuals to benefit from this technology is the price and adaptability of many of the devices currently on the market. To the best of our knowledge, the SuitX Phoenix is the first commercially available exoskeleton that utilizes a semi-passive knee design to overcome some of these problems. The purported advantage of such a design is that it negates the need for additional knee actuators, while still allows the unpowered knee joints to support the patient during stance phase, and clear the ground during the swing phase. The unpowered knee design allows for lighter hardware and a substantially lower cost than exoskeletons with powered knees.

#### Safety and Efficacy of the Phoenix

In this population of thoracic and lumbar spinal cord injury patients, the Phoenix was found to be safe in allowing patients with SCI levels from T7 to L5 to perform functional standing and walking tasks with a trained companion, and patients with SCI levels from T4 to T6 to perform functional tests and walking in a rehabilitation setting. All participants were able to ambulate with the exoskeleton within a reasonable training time.

The Phoenix was found to be efficacious in allowing paraplegic patients to achieve independence on all transitional movements and walking tasks, including on all surface walk tests conducted on carpet and concrete.

The study resulted in no serious adverse events or falls.

#### Walking Speed

This particular cohort of patients had an average walking speed of 0.1–0.15 m/s, which is less than what is reported as the average in the medical literature. In a recent meta-analysis of 14 studies looking into gait speed for paraplegic exoskeleton walkers, the mean speed attained by 84 participants was 0.26 m/s (2). Four variables were found in the majority of studies which might influence gait speed in non-ambulatory individuals using the exoskeleton device to walk: age, injury duration, injury level, and the number of training sessions. While we do not have a definite explanation for the overall slower walking speed compared to the average reported in the literature, we hypothesize that several factors may have influenced this, including the training protocol which was not structured to encourage fast walking, the trainers' (and patients') relative unfamiliarity with a completely novel system, and the relatively high number of mid-high thoracic lesions (47.5% were T4-9 level injuries) and complete SCI (60% of all tested patients were ASIA A) in our series. In the future, more studies are needed on the effect of this particular robot design on the gait speed.

#### User Experience

As a group, all study participants reported moderate to high levels of comfort and satisfaction whilst using the device. Using the Pearson correlation method, we detected a weak negative correlation between the neurological level of injury and rate of perceived exertion, with lower neurological levels correlating with less perceived exertion. The participant experience survey results showed that at all sites, there was an above-average improvement in bowel and bladder movement during use of the Phoenix. Although the study did not detect any statistically significant change in affect, there was an upward trend noted for the positive affect scores and a downward trend for the negative scores. Trends in reported pain scores compared between pre- and post-session showed that the training did not result in any increased pain or discomfort.

#### Comparison With Established Exoskeletons

The Phoenix trial results indicated good performance relative to the Indego trial (21) which included an approximately equivalent number of participants (*n* = 45). The Indego trial was longer with a duration of 8 weeks vs. a mean duration of 24 days in the current study. The FIM score for ambulation indoors was 4.3 (±0.5) for the Indego trial—results from this study indicate a slightly better mean FIM score of 4.6 (±0.5) with Phoenix. The average speed for Indego participants was 0.37 m/s (±0.09 m/s), and in our study was 0.12 m/s (±0.06 m/s). The final mean WISCI-II score for the Indego was 6.8 (±1.5), and 8.6 (±2.2) for the Phoenix. Based on these findings, use of the Phoenix was correlated with a higher level of functional independence and less assistance required for ambulation, but slower walking speeds. This comparison is purely observational and was not based on formal statistical analysis. Furthermore, both studies included relatively small sample sizes, therefore larger sample sizes may provide more precise and accurate comparisons. Future research should include comparing results from this study to additional exoskeletons.

### Limitations of This Study

There were several limitations to this study. Firstly, this was an open and non-blinded / non-controlled study, therefore selection bias toward physically fit and cooperative patients was present. However, due to the severity of paraplegia in all the participants, the bias in the interpretation of the device efficacy is highly unlikely. Secondly, there was a higher proportion of mid-to-upper thoracic level injuries (46.5% T4-9 level), and a high number of complete injury (60%) participants. This distribution may have affected the overall results in subgroup comparisons. Finally, physical parameters such as light touch, proprioception, and spasticity were not analyzed in our cohort and these may also have contributed somewhat to the ability of the patient to utilize the exoskeleton.

## Conclusion

This is the first study to examine the safety and efficacy of a novel lightweight exoskeleton utilizing a semi-passive knee design for ambulation by individuals with paraplegia. We have shown that the Phoenix is efficacious in allowing patients to perform all walking tasks and transitional movements and comparable to technologies currently available on the market. This study also demonstrated a high safety of the device, with no falls, no serious adverse events, and few minor complaints.

The overall user satisfaction was positive, and a trend toward improved psychology was observed. In addition to an acceptable degree of functional independence, participants also reported minimal pain. Although fatigue and exertion levels were reported to be higher after study sessions, those appeared to improve throughout the study period as participants gained experience. This improved stamina occurred over ~4 weeks on average. These findings suggest that long-term training and practice with the exoskeleton could reduce perceived fatigue and exertion.

We believe that the Phoenix exoskeleton, with its increased affordability and improved convenience for everyday usage, will become an important tool for individuals with paraplegia not only to address their life-long exercise needs, but also to use for recreation and health maintenance. We look forward to seeing healthcare systems around the world gradually adopt this and similar technologies into the life-long health maintenance programs for individuals with paraplegia. We believe that it is equally important for agencies designing public infrastructure such as public housing, transportation, and disabled-access, to be aware that this type of technology has now become more ubiquitous in our societies, so that appropriate design elements can be incorporated in long-term city planning.

We recommend further dedicated studies be carried out in the future to examine the applicability in a home or community setting, as well as secondary biophysical changes relating to cardiovascular function, bone mineral density, spasticity level, and neuropathic pain after a longer period of usage by individuals with paraplegia.

## Data Availability Statement

The raw data supporting the conclusions of this article will be made available by the authors, without undue reservation.

## Ethics Statement

The study was reviewed and approved by the respective institutional review boards of all 3 study sites: HKU/HA HKW IRB (Approved IRB# UW 17-169). St. David's Medical Center (Approved IRB# 17-12-02). Ethical and Independent (E&I) Review Service (Approved IRB# 00007807).

## Author Contributions

PK, AV, YJ, and JL were the principle investigators of the three research sites, respectively. PK and AC were responsible for the preparation of the manuscript. YW, KC, and HK were senior authors supervising the research study in Hong Kong and the United States, respectively. All authors contributed to the article and approved the submitted version.

## Conflict of Interest

The study was supported, in part, by US Bionics (d.b.a. SuitX). The SuitX Phoenix devices used in this study were provided by the study sponsor US Bionics. YJ, MM, and HK own rights to the intellectual property and patent(s) of the SuitX Phoenix Medical Exoskeleton. The body of this manuscript was written and edited by authors AC and PK (corresponding author), and both authors declare no conflict of interest toward the results of the study. The results of this study were presented at the 2019 International Spinal Cord Society Annual Scientific Meeting (abstract). AV, YJ, and MM were employed by company US Bionics at Emeryville. The remaining authors declare that the research was conducted in the absence of any commercial or financial relationships that could be construed as a potential conflict of interest.
